# An institutional approach to support the conduct and use of health policy and systems research: The Nodal Institute in the Eastern Mediterranean Region

**DOI:** 10.1186/s12961-015-0032-9

**Published:** 2015-10-01

**Authors:** Fadi El-Jardali, Shadi Saleh, Rawya Khodor, Raeda Abu Al Rub, Chokri Arfa, Habiba Ben Romdhane, Randah R. Hamadeh

**Affiliations:** Department of Health Management and Policy, American University of Beirut, Riad El Solh, PO Box 11–0236, Beirut, 1107 2020 Lebanon; Knowledge to Policy (K2P) Center, Faculty of Health Sciences, American University of Beirut, Riad El Solh, Beirut, 1107 2020 Lebanon; Center for Systematic Reviews of Health Policy and Systems Research (SPARK), American University of Beirut, Riad El Solh, PO Box 11–0236, Beirut, 1107 2020 Lebanon; Research, Advocacy and Public Policy-making, Issam Fares Institute for Public Policy and International Affairs, American University of Beirut, Riad El Solh, PO Box 11–0236, Beirut, 1107 2020 Lebanon; Department of Clinical Epidemiology and Biostatistics, McMaster University, CRL-209, 1280 Main St. West, Hamilton, ON L8S 4 K1 Canada; Collaborative for Leadership and Innovation in Health Systems, Department of Health Management and Policy, Faculty of Health Sciences, American University of Beirut, Riad El Solh, PO Box 11–0236, Beirut, 1107 2020 Lebanon; College of Nursing, Jordan University of Science and Technology, Irbid, 22110 Jordan; National Institute of Labor and Social Studies (INTES), University of Carthage, Tunis, Tunisia; Cardiovascular Diseases Research Laboratory, University of Tunis El Manar, Tunis, Tunisia; Department of Family and Community Medicine, College of Medicine and Medical Sciences, Arabian Gulf University, Manama, Bahrain

**Keywords:** Capacity building, Eastern Mediterranean Region, Evidence-informed policy, Health policy and systems research, HPSR institutional capacity assessment, Nodal Institute

## Abstract

**Background:**

The use of health policy and systems research (HPSR) to support decision making in health systems is limited in the Eastern Mediterranean Region (EMR). This is partly due to the lack of effective initiatives to strengthen regional HPSR capacities and promote its use in decision making. This paper offers a structured reflection on the establishment and core functioning of a HPSR Nodal Institute for the EMR with specific focus on the approach used to support the conduct and use of HPSR. It seeks to gain better understanding of the activities conducted by the Nodal Institute, the methods by which the Nodal Institute implemented these activities, and the outcomes of these activities.

**Methods:**

A multi-faceted approach was implemented by the Nodal Institute in collaboration with regional academic/research institutions, Sub-Nodes. The overall approach was a phased one that included the selection of Sub-Nodes, mapping of academic/research institutions in the EMR, stakeholders’ meetings, and HPSR capacity building workshops, and culminated with a regional meeting.

**Results:**

The mapping of academic/research institutions in the EMR resulted in the identification of 50 institutions, of which only 32 were engaged in HPSR. These institutions have the highest HPSR involvement in information/evidence (84%) and the lowest in human resources for health (34%). Their main HPSR focus areas included quality of healthcare services, patient safety, management of non-communicable diseases, and human resources for health. Regional HPSR challenges among these institutions were identified. The validation and ranking questionnaires resulted in the identification of country-specific HPSR priorities according to stakeholders in three countries. From these results, cross-cutting HPSR priorities among the countries related to primary healthcare, non-communicable diseases, human resources for health, as well as cross-cutting HPSR priorities among stakeholders and according to stakeholders of the countries, were extracted.

**Conclusion:**

The Nodal Institute in the EMR is a promising initiative to support the conduct and use of HPSR in health policies. The approach and findings reported in this paper allow for the development of opportunities towards the building of capacity for HPSR in the region and other countries and provide a roadmap for academic/research institutions interested in HPSR in the region.

## Background

During the past decade, the importance of research in supporting healthcare systems has been highlighted in multiple strategic initiatives. In 2004, the World Health Organization’s (WHO) World Report on Knowledge for Better Health [1] stressed on the need to link research to action. In that same year, the ministerial meetings in Mexico City announced the importance of research for improved health and related systems [2]. Further, in 2008, the global ministerial forum on research for health, held in Bamako, called for linking evidence to policymaking and building research capacity [1]. These initiatives, focusing on health policy and systems research (HPSR), garnered a great attention in the health system field. Defined as the “*the production of knowledge and applications to improve how societies organize themselves to achieve health goals, including how they plan, manage and finance activities to improve health, as well as the roles, perspectives and interests of different actors in this effort,*” HPSR became a key field of interest [3]. Operationally, HPSR integrates research in one or more of the health system building blocks which are defined by WHO and include leadership and governance, health systems financing, human resources for health, service delivery, medical products and technology, and information and evidence [4]. The increasing demand for HPSR has led to the holding of three global symposiums on health systems research. The last symposium, held in Cape Town in 2014, reinforced main action themes for HPSR and recommended capacity development for the strengthening of this field [5].

In low- and middle-income countries (LMICs), HPSR is currently growing in prominence. The Commission on Health Research for Development recommended LMICs to reinforce health research to meet national needs. It was proposed that governments should apportion no less than 0.1% of the annual national health expenditure to HPSR. Nevertheless, only a small fraction out of this suggested standard was estimated to be spent in these countries [6]. It has also been shown that funding for HPSR mostly comes from external sources [7]. However, the main reason behind the low levels of HPSR in LMICs is not exclusively due to unavailability of funds but rather to the weak HPSR capacity of most institutions, including academic/research institutions. A mapping exercise, performed through semi-structured qualitative interviews, in 26 LMICs found that the evidence-informed decision-making culture is very weak in approximately one third of the involved countries [8]. Moreover, an institutional capacity assessment of seven public health schools in East and Central Africa revealed that none of these schools offer degree programs specific to health systems research. Although key strengths in curricula design were present, common constraints related to limited staff competencies, outdated curricula, face-to-face delivery approaches, and restricted access to materials were identified [9]. In addition, an assessment of capacity for HPSR and analysis in seven African universities performed by the Consortium for Health Policy and Systems Analysis in Africa showed that all these universities have varying capacity assets for HPSR and analysis teaching and research, and these should be related to different capacity needs at the individual, organizational, and wider system levels [10]. Moreover, in most LMICs, it has been shown that new established institutions are managed by relatively inexperienced researchers and their growth generally involves the recruitment of less qualified researchers [6]. Furthermore, the main stakeholders are not effectively engaged at the institutional level for policies and program development. Noteworthy, stakeholders in most cases have different agendas and donors dictate research priorities in many instances [6]. This leaves the HPSR field with a restricted range of actors, limited influence at policy levels, and low production of relevant databases and publications [11].

## Context

In the Eastern Mediterranean Region (EMR), health systems research is not adequately developed to be used as evidence for the support of decision making in health systems. Data on national health research systems in 10 EMR countries revealed that only a few countries have a formal national health research system in place [12]. A contributing factor may be linked to the minimal presence of activities required for responsive and needs-oriented health research systems [12]. A study from the region reported that policymakers viewed that research evidence is not being delivered promptly (40.1%) and is not given enough value (35.9%) [13]. A further study performed interviewing researchers who published relevant HPSR showed major barriers for the use of evidence in health policymaking, including insufficient policy dialogue opportunities and collaboration between researchers and policymakers and stakeholders (67.9%), practical constraints to implementation (66%), and non-receptive policy environments (61.3%) [14]. Furthermore, a stocktaking exercise on HPSR production gaps in 12 EMR countries revealed significant inconsistencies between HPSR produced and regional HPSR priorities, emphasizing the need for more HPSR and its alignment with the demand for evidence by policymakers [15]. Additionally, a regional situational analysis exercise showed major gaps in the production of systematic reviews to address identified health policy priorities [16]. The limited number and scope of publicly accessible data was also shown to hinder the ability to employ evidence-informed decisions [17]. Lately, the need to address existing regional HPSR gaps has been growing. However, there has been no real capacity building for HPSR. Capacity building necessitates the integration of efforts at the individual, team, institutional, and systems level [18]. Bennett et al. [7] suggested that institutional HPSR capacity building through HPSR supportive and sustainable homes need to be developed in order to build the field for HPSR. Recently, the Implementation Research Platform and the Alliance for Health Policy and Systems Research have exerted significant efforts on the building of institutional capacities for HPSR by establishing four academic/research institutions engaged in HPSR, called Nodal Institutes, in selected regions. One of these Nodal Institutes has been formed in the EMR with the main objective of facilitating the conduct and utilization of relevant HPSR among academic/research institutions and allowing these institutions to build team and individual capacities within their areas of work. Since then, the Nodal Institute has engaged several academic/research institutions, called Sub-Nodes. The role of these Sub-Nodes is to promote HPSR in their own country. In collaboration with the Sub-Nodes, the Nodal Institute has achieved its main objectives through the implementation of various activities. To our knowledge, this institute constitutes one of the first HPSR institutional capacity building initiatives in the EMR.

This paper offers a structured reflection on the core functioning of the Nodal Institute since its establishment with a specific focus on the approach used to support the conduct and use of HPSR in the EMR. It seeks to gain a better understanding of the (1) activities conducted by the Nodal Institute, (2) the methods by which the Nodal Institute implemented these activities, and (3) the outcomes of these activities, including the regional areas of involvement of academic/research institutions in HPSR, regional HPSR focus areas and challenges, and country-specific HPSR priorities in three EMR countries.

## Methods

**Figure 1:**
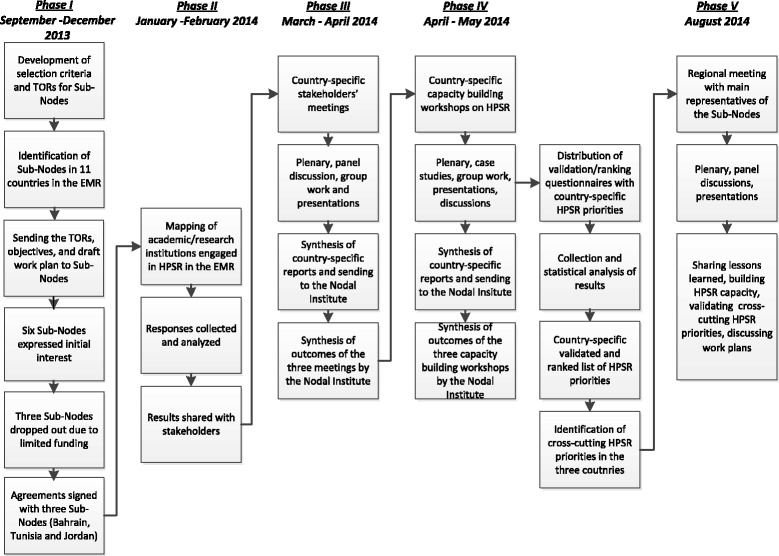
Key health policy and systems research activities conducted during the first year of the study.

A multi-faceted approach was implemented by the Nodal Institute in collaboration with regional academic/research institutions (Figure 1). The overall approach used a combination of qualitative and quantitative research methods. The designated Nodal Institute in the EMR, served by a core research team from the Faculty of Health Sciences at the American University of Beirut, led and coordinated all aspects of the initiative in collaboration with the selected Sub-Nodes in the region.

The Sub-Nodes were selected in phase I based on pre-defined eligibility criteria which included being an academic or a research institution, located in the EMR, involved in HPSR, and being able and willing to deliver on committed activities. The Nodal Institute balanced between the countries of the Sub-Nodes by considering differences in their geographic distribution, health systems, and economic context. After developing the Terms of Reference, 11 academic/research institutions located in Bahrain, Egypt, Jordan, Iran, Kuwait, Morocco, Palestine, Sudan, Tunisia, United Arab Emirates, and Yemen were contacted through initial letters of invitation. Six of these institutions expressed initial interest and three were selected based on the availability of funds and the readiness of their research teams. Successively, the Terms of Reference with the expected objectives and a draft work plan were sent and agreements were finalized with three Sub-Nodes in Bahrain, Jordan, and Tunisia.

The first activity of the Nodal Institute within phase II included a mapping activity in order to gather data about the academic/research institutions engaged in HPSR in the EMR*.* With inputs from the Sub-Nodes, a mapping questionnaire was developed and sent to academic/research institutions for the collection of their responses over a 2-month period. This mapping questionnaire is composed of one closed-ended and two open-ended questions. The closed-ended question was designed based on WHO’s defined health system building blocks, in which HPSR can be involved, in order to identify areas of involvement of institutions in HPSR. Findings from this question were analyzed by the Nodal Institute using quantitative analysis. The two open-ended questions were designed to identify current regional HPSR focus areas and challenges. Findings from these two questions were analyzed using thematic analysis, from which recurring themes across the academic/research institutions emerged.

In phase III, and once regional HPSR areas of involvement of academic/research, HPSR focus areas, and challenges had been identified, three national stakeholders’ meetings, one in each of the three Sub-Node countries, were conducted in order to initiate the development and strengthening of HPSR capacity in the region. According to a sampling frame that was adapted and employed in previous studies in the region [19], the selection criteria of a wide range of key stakeholders of the meetings and trainings of the Nodal Institute was identified. Around 25–30 participants, including researchers, policymakers, decision-makers, civil society groups, and funders were present in each of three Sub-Node meetings. These national meetings, which were held for one day, introduced HPSR and helped in understanding the challenges in evidence-informed policy. They also helped in identifying the main opportunities to endorse HPSR in the region. Additionally, ways on how to translate research into policy interface were highlighted. In these meetings, deliberations over the main HPSR areas which need further research resulted into agreed upon main HPSR priorities for each of the three countries.

In phase IV, and on the basis of the reports from stakeholder meetings, three national capacity building trainings on HPSR were spanned over 2 days and adapted according to each country based on its needs and priorities. Around 30 participants, including researchers, students, policymakers, and practitioners in the HPSR field, joined each of these three trainings. The trainings provided a definition of HPSR, and a situational analysis of HPSR in the region. They also highlighted how HPSR can strengthen health systems, policymaking, scale-up interventions, and how it can help in enhancing the use of evidence by decision makers. Sessions on the process of converting HPSR problems/priorities into research, knowledge translation mechanisms, and steps for future work were also introduced. Moreover, in order to get a final validated and ranked list of country-specific HPSR priorities, the Nodal Institute compiled the main country-specific HPSR priorities for each of Bahrain, Jordan, and Tunisia (which were agreed upon during the stakeholders’ meeting) into three validation and ranking questionnaires and distributed them to participants during the three trainings. These country-specific priorities were ranked based on a sampling framework that was developed by Varkevisser et al. [20] and further customized and used in a nine country study in the region by El-Jardali et al. [21].

- Relevance: of the research priorities to policy concerns?
- Urgency: are they needed within the next 3–5 years?
- Feasibility: are the research priorities achievable in your country?
- Applicability: once we have evidence on these research priorities, can they drive policy changes?
- Originality: has this priority not already been addressed in your country?

Each of these criteria was ranked on a 3-point Likert scale (1 = low, 2 = medium, and 3 = high). Total scores were computed and all criteria were given equal weights. Means and standard deviations for scores of each of the five criteria were computed. The five country-specific HPSR priorities with the highest mean scores were chosen as top country-specific HPSR priorities. The three country-specific HPSR priorities with the lowest mean scores were chosen as bottom country-specific HPSR priorities. Based on these results, the Nodal Institute identified cross-cutting HPSR priorities among the three countries.

In addition, country-specific HPSR priorities were further weighted according to the different types of stakeholders that were present during the trainings. The means and standard deviations were computed for the country-specific HPSR for each type of stakeholders. The three country-specific HPSR priorities with the highest mean scores were designated as top country-specific HPSR priorities for each type of stakeholders. Based on these results, the Nodal Institute identified cross-cutting HPSR priorities among the different types of stakeholders in each country (Bahrain, Jordan, and Tunisia), and cross-cutting HPSR priorities according to the type of stakeholders in all three countries.

Once country-specific work was complete, the last phase of the Nodal Institute’s activities in its first year of implementation, phase V, included a 2-day regional meeting for the three representatives of the Sub-Nodes (Bahrain, Jordan, and Tunisia). The Sub-Nodes presented their country-specific activities, emphasizing their main lessons learned. Then, the results of the mapping activity and the validation and ranking questionnaires were discussed. Additionally, all cross-cutting HPSR priorities were validated. Afterwards, the Nodal Institute with the Sub-Nodes discussed their work plans for upcoming years.

## Results

The following sections present the results of the mapping of regional academic/research institutions involved in HPSR and the results of the validation and ranking questionnaires. The results of the mapping of regional academic/research institutions involved in HPSR present the findings of three main issues: (1) regional areas of involvement of academic/research institutions in HPSR, (2) commonly identified regional HPSR focus areas, and (3) commonly identified regional HPSR challenges. A total of 50 academic/research institutions were mapped, of which only 32 were engaged in HPSR.

### Regional areas of involvement of academic/research institutions in HPSR

**Figure 2:**
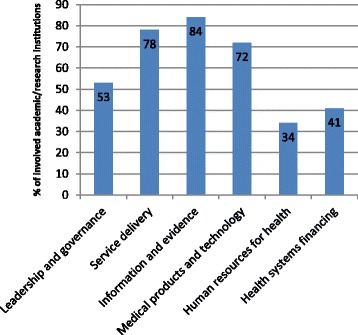
Areas of involvement of academic/research institutions in health policy and systems research.

The mapping of academic/research institutions in the EMR revealed various areas of involvement in HPSR (Figure 2). The involvement of regional academic/research institutions in HPSR was the highest in areas of information and evidence and service delivery, at 84% (27 institutions) and 78% (25 institutions), respectively. More than two-thirds of the institutions (72%) were engaged in medical products and technology and 53% in leadership and governance. Approximately one-third of the institutions were found to be concerned with human resources for health (34%) and health systems financing (41%). Additional areas of involvement of regional academic/research institutions in HPSR were further highlighted. Some institutions were highly involved in advocacy and development. Furthermore, a small number of institutions mentioned priority setting and knowledge translation as part of their areas of involvement in HPSR.

### Commonly identified regional HPSR focus areas among academic/research institutions

Based on the results of the mapping activity that was conducted with regional academic/research institutions, a range of HPSR focus areas emerged. The following themes were the main emergent themes in terms of frequency: quality of healthcare services, patient safety, management of non-communicable diseases, and human resources for health. Research on insurance systems, such as universal health coverage as well as on the utilization of healthcare services, was also frequently mentioned. In addition, HPSR was being conducted on the evaluation of health programs and policies and their effects on health outcomes. These regional academic/research institutions indicated research on health systems (in terms of their development and reforms, and in times of conflict settings) and health financing as part of their research agendas.

### Commonly identified regional HPSR challenges among academic/research institutions

The mapping activity also identified a set of challenges concerning HPSR. The regional academic/research institutions indicated the lack of incentives for partnerships between various stakeholders as a major challenge for HPSR. In several academic/research institutions of different countries, the high presence of poor research environments and problems in knowledge translation and dissemination of findings also thematically emerged as challenges. Additionally, challenges including weak data systems with scattered health information and lack of access to reliable data were highlighted. The low interest in evidence-informed policy and the lack of priority setting in addition to the lack of funding were also acknowledged as challenges across a number of regional institutions. Finally, the issues of lack of adequate research workforce, and the lack of collaborations between different institutions also arose.

The results of the validation and ranking questionnaires present the findings of two main issues: (1) country-specific HPSR priorities and (2) country-specific HPSR priorities according to the type of stakeholders.

### Country-specific HPSR priorities in three EMR countries

Based on the validation and ranking questionnaires distributed to each of Bahrain, Jordan, and Tunisia, the main country-specific HPSR priorities were identified. As discussed in the methods section, the top five country-specific HPSR priorities with the highest mean scores were chosen as top country-specific HPSR priorities and the those with the lowest mean scores were chosen as bottom HPSR priorities for each of the three countries. The results are shown in Table 1.

**Table 1:** **Health policy and systems research priorities ranking in Bahrain, Jordan, and Tunisia**

**Country**	**Highest priorities**	**Rank**	**Mean (SD)**	**Lowest priorities**	**Rank**	**Mean (SD)**
Bahrain (n = 17)	Continuous assessment of the quality of services	1	2.58 (0.26)	Research on the existing health system building blocks	1	2.34 (0.33)
	National health-related policies	2	2.51 (0.25)	Identification of health topics of national, regional, and global importance	2	2.31 (0.41)
	Accessibility to health services	3	2.46 (0.57)	Succession planning	3	2.31 (0.41)
	Cost effective budget allocation	4	2.44 (0.52)			
	Sustainability – maximum value of money spent	5	2.44 (0.53)			
Jordan (n = 14)	Rational drug use	1	2.74 (0.37)	Universal health insurance	1	2.42 (0.34)
	Migration of qualified healthcare providers	2	2.73 (0.38)	Regulation of private health sector	2	2.39 (0.74)
	Primary healthcare	3	2.71 (0.20)	Out-of-pocket health expenditure	3	2.25 (0.38)
	Retention of healthcare providers especially in remote areas	4	2.71 (0.40)			
	Non-communicable disease management	5	2.69 (0.32)			
	Health management information system	6	2.69 (0.38)			
Tunisia (n = 18)	Primary healthcare	1	2.53 (0.36)	Coherency between what is declared and what is done	1	2.02 (0.39)
	Maternal and child health	2	2.41 (0.32)	Universal coverage	2	2.01 (0.53)
	Health system governance	3	2.35 (0.43)	Complementarity between public and private sector	3	1.96 (0.40)
	Non-communicable disease prevention and control	4	2.34 (0.33)			
	Sexually transmitted diseases and HIV	5	2.31 (0.41)			
	Human resources for health: training	6	2.31 (0.41)			

By matching top HPSR priorities across the three countries, the Nodal Institute identified three cross-cutting HPSR priorities, namely primary healthcare, non-communicable diseases (prevention, management, and control), and human resources for health (training, retention of healthcare providers, migration of qualified healthcare providers). These priorities were validated during the regional meeting.

### Country-specific HPSR priorities according to the type of stakeholders in three EMR countries

As discussed in the methods section, the three country-specific HPSR priorities with the highest mean scores were designated as top HPSR priorities for each type of stakeholders (Table 2).

**Table 2:** **Highest health policy and systems research priorities according to stakeholder type in Bahrain, Jordan, and Tunisia**

	**Stakeholders**	**Highest priorities**
Bahrain	Policymaker	1. Cost effective budget allocation
		2. Continuous assessment of the quality of services
		3. Accessibility to healthcare services
	Academia/Researcher	1. Sustainability – maximum value of money spent
		2. Succession planning
		3. National health-related policies
	Others	1. Cost effective budget allocation
		2. Continuous assessment of the quality of services
		3. Accessibility to healthcare services
Jordan	Policymaker	1. Primary healthcare
		2. Non-communicable disease management
		3. Violence against healthcare providers
	Academia/Researcher	1. Violence against healthcare providers
		2. Universal health insurance
		3. Decentralization of healthcare system
	Representative of a non-governmental association	1. Primary healthcare
		2. Non-communicable disease management
		3. Violence against healthcare providers
	Others	1. Non-communicable disease management
		2. Violence against healthcare providers
		3. Universal health insurance
		4. Decentralization of healthcare system
Tunisia	Policymaker	1. Health financing
		2. Accessibility to healthcare services
		3. Human resources for health: mobilization
	Academia/Researcher	1. Coherency between what is declared and what is done
		2. Health financing
		3. Accessibility to healthcare services
		4. Human resources for health: mobilization
	Representative of a non-governmental association	1. Accessibility (healthcare pathway)
		2. Human resources for health: mobilization
		3. Coherency between what is declared and what is done
	Others	1. Coherency between what is declared and what is done
		2. Complementary between public and private sector
		3. Primary healthcare

By matching top HPSR priorities among the different types of stakeholders in each of the three countries, the Nodal Institute identified cross-cutting HPSR priorities among the different stakeholder types in each country, as indicated below. These priorities were validated during the regional meeting.

*Bahrain*

- Cost effective budget allocation
- Continuous assessment of the quality of services
- Accessibility to healthcare services

*Tunisia*

- Health financing
- Accessibility to healthcare services
- Human resources for health: mobilization
- Coherency between what is declared and what is done

*Jordan*

- Primary healthcare
- Non-communicable disease management
- Violence against healthcare providers
- Universal health insurance
- Decentralization of healthcare system

By matching top HPSR priorities according to the type of stakeholders in the three countries, the Nodal Institute identified policymakers (regarding accessibility to healthcare services) and academia/researchers (regarding health financing, including sustainability of the healthcare system) as cross-cutting HPSR priorities according to stakeholder types in the three countries. These priorities were validated during the regional meeting.

In addition, by deliberating over the identified HPSR priorities during the regional meeting, each of the representatives of the three Sub-Nodes interestingly identified different mechanisms to translate the identified HPSR priorities into specific research questions in order to address evidence-based decision making.

For example, an institutional research agenda will be considered in the Sub-Node in Bahrain as a mechanism to ensure the translation of the identified HPSR priorities into researchable questions.

“*Our institution will incorporate the identified HPSR priorities into its institutional research agenda. Researchers who seek funding from our institution and focus on a HPSR priority from this agenda will be given priority for funding.*” (Key representative of the Sub-Node in Bahrain).

As for the Sub-Node in Jordan, a health policy forum has been developed and will be used as a specific strategy to address the identified HPSR priorities.

“*The health policy forum in our institution, which consists of policy makers and researchers, will formulate a work plan for translating the identified HPSR priorities into research questions that will be published and disseminated to key stakeholders. Researchers of this forum will develop proposals and conduct research studies on the identified HPSR priorities.*” (Key representative of the Sub-Node in Jordan).

The Sub-Node in Tunisia, and as part of a scale-up strategy, will focus on one of the identified HPSR priorities.

“*Our institution will start focusing on producing research evidence on non-communicable diseases prevention and control, as it is one of our top HPSR priorities. In addition, we will translate this research evidence and promote its use in policy and practice.*” (Key representative of the Sub-Node in Tunisia).

## Discussion

### Principal findings

By applying a multi-phased approach and involving different stakeholders, the study identified the main areas of involvement of regional academic/research institutions in HPSR, regional HPSR focus areas and challenges, and country-specific HPSR priorities in Jordan, Tunisia, and Bahrain.

The results of the mapping activity showed no HPSR alignment between the regional areas of involvement of academic/research institutions in HPSR and their HPSR focus areas. Although the most common areas of involvement of these academic/research institutions were reported to be in information and evidence and service delivery, these institutions have been mainly found to be focusing their HPSR on the quality and utilization of healthcare services and the management of non-communicable diseases. This may be attributed to the HPSR challenges that were identified among these regional institutions which mainly include poor HPSR research environments, lack of availability and reliability of HPSR data, inadequate knowledge translation and priority setting mechanisms, and a lack of funding and research workforce.

Based on the results of the validation and ranking questionnaires, the cross-cutting HPSR priorities among the different stakeholders in Jordan, Tunisia, and Bahrain demonstrated important fluctuations in the prioritization of HPSR when compared to the country-specific HPSR priorities without further weighing according to the type of stakeholders. In Jordan, universal health coverage was one of the most important cross-cutting HPSR priorities among some stakeholders, however, this HPSR priority was one of the least important when not weighing according to the type of stakeholder. Furthermore, although HPSR on violence against healthcare providers and the decentralization of healthcare systems were identified as cross-cutting HPSR priorities among the different types of stakeholders in Jordan, they were not among the highest country-specific HPSR priorities. It is important to note that HPSR on primary healthcare and the management of non-communicable diseases were found to be HPSR priorities in Jordan regardless of weighing according to stakeholders. Similarly, in Tunisia, three HPSR priorities which were health financing, accessibility to healthcare services, and human resources for health in terms of the provision of trainings were cross-cutting HPSR priorities among the stakeholders. However, these areas of HPSR were not identified as one of Tunisia’s highest priorities when not weighing according to stakeholders. This also applies to the HPSR priority on cost effective budget allocation in Bahrain. HPSR priorities in Bahrain focused on the continuous assessment of the quality of services and the accessibility to healthcare services regardless of weighing according to stakeholders. These discrepancies reflect the different interests of the key stakeholders in HPSR, which may be due to different professional backgrounds, current and highlighted issues of the country, and areas of involvement of these stakeholders in HPSR in the different EMR countries.

The cross-cutting HPSR priorities according to the type of stakeholders of the three countries further demonstrate that different types of stakeholders have different interests in HPSR. Policymakers in Jordan, Tunisia, and Bahrain held accessibility to healthcare services as their main HPSR priority, despite having other HPSR priorities. This may reflect that policymakers are more interested in knowing which strategies are required to improve the accessibility to healthcare and to produce a greater impact on the outcomes delivered by their health systems. On the other hand, academicians and researchers in the three countries held health financing as their main HPSR priority. This may be related to the fact that healthcare costs for academicians and researchers are majorly considered an important aspect of the efficiency and effectiveness of healthcare systems, especially when it comes to the sustainability of health systems. Furthermore, the fact that non-governmental associations in the three countries have no cross-cutting HPSR priorities may reflect important differences in the HPSR agenda of their donors. Noteworthy, the fluctuation in these cross-cutting priorities may also indirectly reflect the emergence of country-specific problems that trigger stakeholders’ interests to specific issues in the region.

### Strengths and limitations

The Nodal Institute is one of the first institutional initiatives to build HPSR capacity at different levels in the EMR. Through the involvement of diverse stakeholder groups, this study served in facilitating the conduct and the utilization of relevant HPSR in the region. It is one of the first initiatives to identify HPSR priorities among various stakeholders in different countries, and more importantly to identify the cross-cutting HPSR priorities in the region. Our study also resulted in the identification of the main challenges in the use and conduct of HPSR in policies and interventions in order to bridge HPSR gaps and enhance HPSR capacity building initiatives.

The multi-phased processes and activities implemented have several strengths. First, the combination of qualitative and quantitative research techniques in the different phases of the study allowed for a more holistic analysis of the different perspectives on HPSR. Second, the grouping of multidisciplinary stakeholders increased the level of interaction as well as the involvement in HPSR. This stronger interaction will allow for more support to scale-up HPSR in the region and to galvanize interests in the field. Third, the HPSR priorities obtained from the activities will encourage researchers to invest more efforts in converting these priorities to specific research questions. Fourth, the regional meeting allowed for the development of work plans for HPSR in the region. The work plans included activities related to the conduct of more HPSR capacity building workshops in the Sub-Nodes, the creation of HPSR groups in each of the Sub-Nodes with main objectives of constructing strong communication networks between researchers and other stakeholders and enabling the identification of emerging HPSR, and convening a regional symposium on HPSR. Finally, our methodology can be simulated in other contexts as a main approach for building or strengthening existing HPSR capacities.

Our study has a number of limitations. First, the views presented on priorities for HPSR represent those of stakeholders present in the three country meetings. Although care has been taken in ensuring wide and representative participation, the priorities nevertheless reflect the opinions of those present. Second, the countries that were part of the initiative represent the socioeconomic and geographic variety of the region. However, the ideas and priorities presented cannot be generalized to all countries given the complex context of the region.

### Findings in relation to previous studies

The findings of this study are congruent with previous findings from LMICs in other regions. A study assessing the production of HPSR in LMICs found the highest number of HPSR publications in areas related to service delivery and highlighted the need to address the imbalance between areas of involvement in HPSR publications and HPSR priorities in order to address knowledge gaps [22]. A previous study performed by Xue et al. [23] also showed a high number of publications in health service delivery and health finance and identified a lack of attention among researchers and implementers on specific HPSR priorities. As for HPSR priorities in LMICs, our findings correspond to some of the priorities identified by policymakers in Nigeria related to the access to medical products and technologies, health service delivery, and shortfalls in the supply of professional personnel [24]. In addition, Ranson et al. [25] highlighted key HPSR priorities for human resources for health in LMICs among key stakeholders. Further, a recent research priority-setting exercise identified main HPSR priorities related to health financing and human resources based on stakeholders in nine LMICs in the Middle East and North Africa region [21].

### Implications for funders, researchers, and policymakers

This structured reflection provides a baseline assessment of the involvement of regional academic/research institutions in HPSR, main HPSR focus areas, and challenges in the EMR. It also provides clear insights into the stakeholders’ HPSR priorities in three countries of the region. The methodology used by this study can be useful for other countries and regions that are planning to build HPSR capacities.

The detected findings could also be used to reduce the significant HPSR gap between the areas of involvement of academic/research institutions in HPSR and HPSR priorities in the EMR. Given the fact that the development of HPSR is impeded by a heavy reliance on international funding for HPSR [7], it is the hope that our findings can attract national and international funding agencies to provide further financial support to address the identified HPSR priorities, thus reducing the HPSR knowledge gap in the EMR. Moreover, main HPSR priorities were identified which can be translated into specific research questions to address evidence. A synthesis of already existing evidence on these HPSR priorities is needed to determine which specific aspects of those priorities are already in the literature and which can be further emphasized by primary studies. The findings can also inform main HPSR stakeholders and can direct future strategic plans and investments towards building HPSR capacity in the region. Finally, this study provides a roadmap for academic/research institutions interested in HPSR on examples of the implementation of HPSR. It also provides insights for other potential Sub-Nodes in the region to effectively implement HPSR.

## Conclusion

The establishment of the Nodal Institute in the EMR is a promising initiative to support the conduct and the use of HPSR in policy and programme planning, implementation, and scale up. By building HPSR capacity in the EMR, this institute will overcome some of the main regional HPSR challenges, which include lack of incentives for partnerships between various stakeholders, weak HPSR expertise capacity, low interest in evidence-informed policy processes, and lack of HPSR priority setting. This HPSR reform initiative allows for the development of opportunities towards the building of capacity for HPSR in the region through strengthening HPSR environments, encouraging collaborations between different stakeholders and institutions, and driving the interests of key stakeholders, including policymakers and national and international funders.

As the Nodal Institute develops more strategic HPSR activities and plans in the subsequent years in the EMR, more efforts and resources towards building HPSR capacity will exhibit a greater support for the conduct and the use of HPSR in regional policies and programs.

## Abbreviations

EMR: Eastern Mediterranean Region
HPSR: Health policy and systems research
LMICs: Low and middle income countries
WHO: World Health Organization
